# Methyljasmonate and salicylic acid contribute to the control of *Tilletia controversa* Kühn, causal agent of wheat dwarf bunt

**DOI:** 10.1038/s41598-020-76210-2

**Published:** 2020-11-05

**Authors:** Ghulam Muhae-Ud-Din, Delai Chen, Taiguo Liu, Wanquan Chen, Li Gao

**Affiliations:** 1 State Key Laboratory for Biology of Plant Disease and Insect Pests, Institute of Plant Protection, Chinese Academy of Agricultural Sciences, Beijing, 100193 People’s Republic of China; 2 College of Plant Protection, Gansu Agricultural University, Lanzhou, 730070 Gansu Province People’s Republic of China

**Keywords:** Developmental biology, Microbiology, Plant sciences

## Abstract

*Tilletia controversa* Kühn (TCK) is the causal agent of dwarf bunt of wheat, a destructive disease in wheat-growing regions of the world. The role of Meja, SA and Meja + SA were characterized for their control of TCK into roots, coleoptiles and anthers. The response of the defence genes PR-10a, Catalase, COI1-1, COII-2 and HRin1 was upregulated by Meja, SA and Meja + SA treatments, but Meja induced high level of expression compared to SA and Meja + SA at 1, 2, and 3 weeks in roots and coleoptiles, respectively. The severity of TCK effects in roots was greater at 1 week, but it decreased at 2 weeks in all treatments. We also investigated TCK hyphae proliferation into coleoptiles at 3 weeks and into anthers to determine whether hyphae move from the roots to the upper parts of the plants. The results showed that no hyphae were present in the coleoptiles and anthers of Meja-, SA- and Meja + SA-treated plants, while the hyphae were located on epidermal and sub-epidermal cells of anthers. In addition, the severity of hyphae increased with the passage of time as anthers matured. Bunted seeds were observed in the non-treated inoculated plants, while no disease symptoms were observed in the resistance of inducer treatments and control plants. Plant height was reduced after TCK infection compared to that of the treated inoculated and non-inoculated treatments. Together, these results suggested that Meja and SA display a distinct role in activation of defence genes in the roots and coleoptiles and that they eliminate the fungal pathogen movement to upper parts of the plants with the passage of time as the anthers mature.

## Introduction

Wheat is a staple food crop and considered an essential element for food security in the world^1,2^. Plants live in complex environments in which they interact with a broad range of microbial pathogens with different lifestyles and infection strategies^3,4^. Dwarf bunt of wheat is a serious wheat disease caused by *Tilletia controversa* Kühn (TCK)^5–7^. TCK is a soilborne and seedborne pathogen, and it infects wheat crops under favourable conditions. The teliospores require several weeks for germination in an unchanging cool temperature obtained by continuous snow cover. After teliospores germination, hyphae infect roots and coleoptiles, and they move to the reproductive parts of the wheat crops^8^. Yield losses due to TCK have reached 70–80% in cold wheat growing areas of the world^9,10^. Symptoms of dwarf bunt infection do not generally manifest until culm elongation of the host. During the seedling stage, leaves of the infected plants might have a characteristic flecking, depending on the wheat cultivar/dwarf bunt race combination. Extreme dwarfing is a characteristic symptom of dwarf bunt, and height reduction ranging from 25 to 66% has been observed. Heads of wheat plants infected with dwarf bunt are often longer, wider, and thicker than heads, and they are generally squarrose. In general, all florets of a single spikelet are filled with bunt balls (sori) with a fishy smell of trimethylamine^11^. The use of traditional fungicides to control dwarf bunt pathogen is restricted, and it increases environmental pollution^12^. Thus, new environmentally friendly chemicals and methods to control dwarf bunt pathogen are needed^13^. Plants have a sound and complex defence system to recognize and respond to pathogen infection^14–16^. Several defence-related actions, including signal generation, pathogen perception, transmission, and stimulation of defence-related chemicals that restrict pathogen invasion, have been classified and identified^2^. The interaction between plant R-genes and pathogen Avr genes are important for initial recognition of the pathogen^17^. In general, recognition of the pathogen by the plant results in increased endogenous levels of nitric oxide (NO), reactive oxygen species (ROS), jasmonic acid (JA), ethylene and salicylic acid (SA), which activate defence responses through various signalling networks^3,18^. Signalling networks activate a continuous series of defence responses that eliminate the aggressiveness of the fungal pathogens^18^. SA plays a key role in disease resistance signalling pathways^19^, and its response is typically more effective against biotrophic pathogens than necrotrophic pathogens^20^. JA is a major defence hormone and significantly increases the resistance level of the plants against fungal pathogens^3,21^. The responses activated by SA, JA, ROS, and NO include upregulation of antimicrobial compounds, phytoalexins, and phenylalanine lyase, an enzyme important for plant defence, hypersensitive response, and deposition of defence material on the cell wall^22^. The molecular responses of roots and coleoptiles to pathogen infections involve several signalling pathways^23^. Induction of specific antimicrobial compounds, i.e., PR proteins, positively correlates with the onset of systematic acquired resistance (SAR) and can be regulated by the exogenous application of SA and Meja^24–26^. Catalase is the key enzyme for plant defence systems and is the major component of oxidative stress metabolism^27,28^. Coronatine-insensitive (*COI*) molecules play a significant role in the defence strategies of plants under stress conditions^29^. COI1 signalling has been associated with basal resistance to the soilborne necrotrophic pathogen, *Pythium ultimum* in *Arabidopsis*^30^. Similarly, HRin1 is the hypersensitive response protein associated with the host hypersensitive defence response^28^. These defence genes increase the defence systems of the roots and coleoptiles against *Pseudomonas fluorescens*, *Pythium* and other fungal pathogens^30,31^.

In the present study, we determined the response of PR-10a, COI, HRin1 and catalase in roots and coleoptiles after exogenous application of Meja, SA and their combination (Meja + SA). Exogenous application of the above inducers of resistance to a highly susceptible cultivar increased the defence level of the highly susceptible cultivar against dwarf bunt of wheat. We measured the expression of defence-related genes in roots and coleoptiles after treatment with inducers of resistance for different time intervals. Moreover, we investigated the invasion of TCK into epidermal and sub-epidermal cells of the anther in treated inoculated (inducer + TCK), non-treated inoculated (TCK) and control plants. Plant height was also measured in all treatments. The results suggested that inducers of resistance play distinct roles in TCK control in the roots and coleoptiles and that they increase the plant height of the wheat crops. To the best of our knowledge, this study is the first to investigate Meja, SA and Meja + SA to activate defence pathways in wheat against TCK.

## Results

### PR-10a, catalase and CoI1-1 transcriptional levels in roots after treatment of wheat plants with resistance inducers

**Figure 1:**
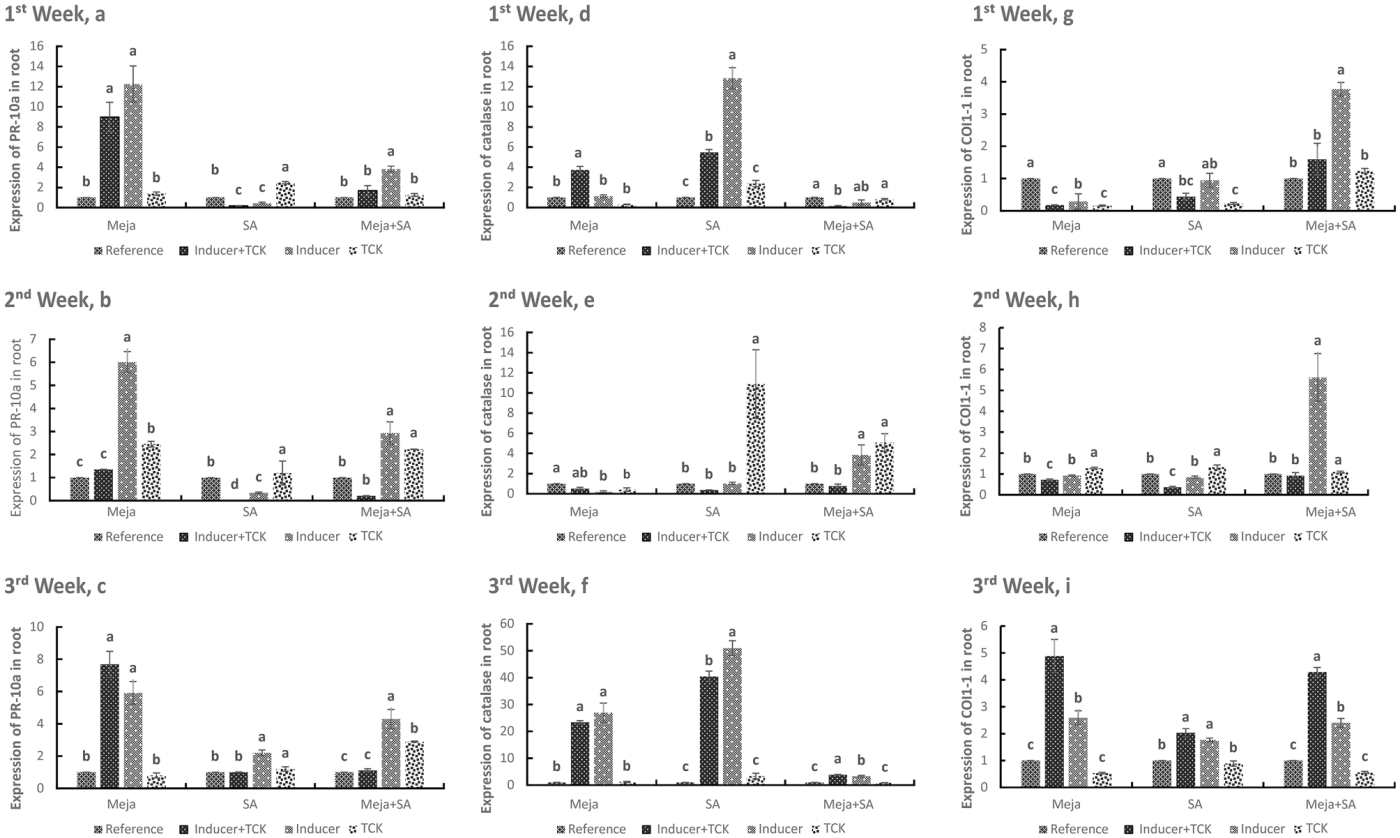
Time course of PR-10a, catalase and C0I1-1 transcript expression in roots in treated inoculated (inducer + TCK), treated non-inoculated (inducer), non-treated inoculated (TCK) plants. Plants receiving no inducer of resistance or TCK inoculation were used as controls. Treated mean application of inducers of resistance and inoculated mean application of TCK are shown. The treatments within the time interval followed by the same letters are not statistically significant using Tukey’s LSD test (*P* < 0.05).

The transcriptional level of PR-10a was analysed by qPCR in the roots of a highly susceptible cultivar (Dongxuan 3) after treatment with inducers of resistance (SA, Meja and Meja + SA). For PR-10a, Meja induced the maximum level of expression at 1 week with a 12.26-fold increase in treated non-inoculated (inducer) plants compared to reference. In treated inoculated (inducer + TCK) plants, an 8.98-fold increase was recorded after Meja treatment, but in the non-treated inoculated (TCK) group, an increase of only 1.39-fold was recorded after treatment with Meja. SA treatment downregulated the expression of PR-10a by 0.18-fold (inducer + TCK) and 0.45-fold (inducer) at 1 week compared to the reference (Fig. 1a). A similar response was recorded after 2 and 3 weeks for Meja and SA (Fig. 1b,c). With regard to Meja + SA, the maximum transcription level of PR-10a was noted at 3 weeks, reaching a 4.29-fold increase compared to the reference in inducer (Fig. 1c), and it reached a 3.84-fold increase compared to the reference at 1 week (Fig. 1a). The above results indicated that the expression of PR-10a was increased at 1 week in the Meja treatment compared to the expression level at 2 and 3 weeks, which may be due to the prevalence of TCK in the roots during the first week compared to the other weeks. The inducers of resistance may have degraded the cell wall of TCK during weeks 2 and 3, or they may have activated the host resistance genes or pathogen avirulence genes, encoding specific elicitor molecules.

The response of catalase to the exogenous application of hormones was comparatively higher compared to the reference. As shown in Fig. 1, catalase expression slowly increased over time. The expression level of catalase increased to 50.95-fold (inducer) after 3 weeks of SA treatment (Fig. 1f), and significant expression was recorded after 1 week and 2 weeks in the inducer and inducer + TCK plants, respectively, for SA (Fig. 1d,e). Similarly, catalase responded in a similar way to the exogenous application of Meja at 3 weeks. The highest expression in the Meja treatment occurred at 3 weeks with an increase of 26.9-fold for inducer plants, which was higher compared weeks 1 and 2 (Fig. 1f). In the Meja + SA treatment, upregulated expression of catalase was recorded in weeks 2 and 3 (Fig. 1e,f), but the catalase expression was reduced in the inducer + TCK and inducer treatments compared to the reference at 1 week (Fig. 1d).

COI1-1 responded similarly as it was downregulated by both Meja and SA after 1 week in all treatments, but a significant upregulation in COI1-1 was noted in the TCK treatment compared to the reference at week 2. The expression of COI1-1 was significantly upregulated after Meja + SA treatment at week 1 in the inducer treatment compared to the reference (Fig. 1g). With regard to Meja, COI1-1 expression was increased by 4.89-fold and 2.59-fold in the inducer + TCK and inducer treatments, respectively, compared to the reference (Fig. 1i). In the SA treatment, COI1-1 expression was increased by 2.03-fold and 1.77-fold in the inducer + TCK and inducer, respectively, compared to the reference. After the Meja + SA treatment, COI1-1 expression was increased by 4.29-fold and 2.4-fold in the inducer + TCK and inducer, respectively, compared to the reference. However, there was no significant difference for the Meja, SA and Meja + SA for TCK treatments compared to their reference at week 3 (Fig. 1i).

### COI1-2 and HRin1 levels in roots and PR-10a level in coleoptiles after treatment of wheat plants with resistance inducers

**Figure 2:**
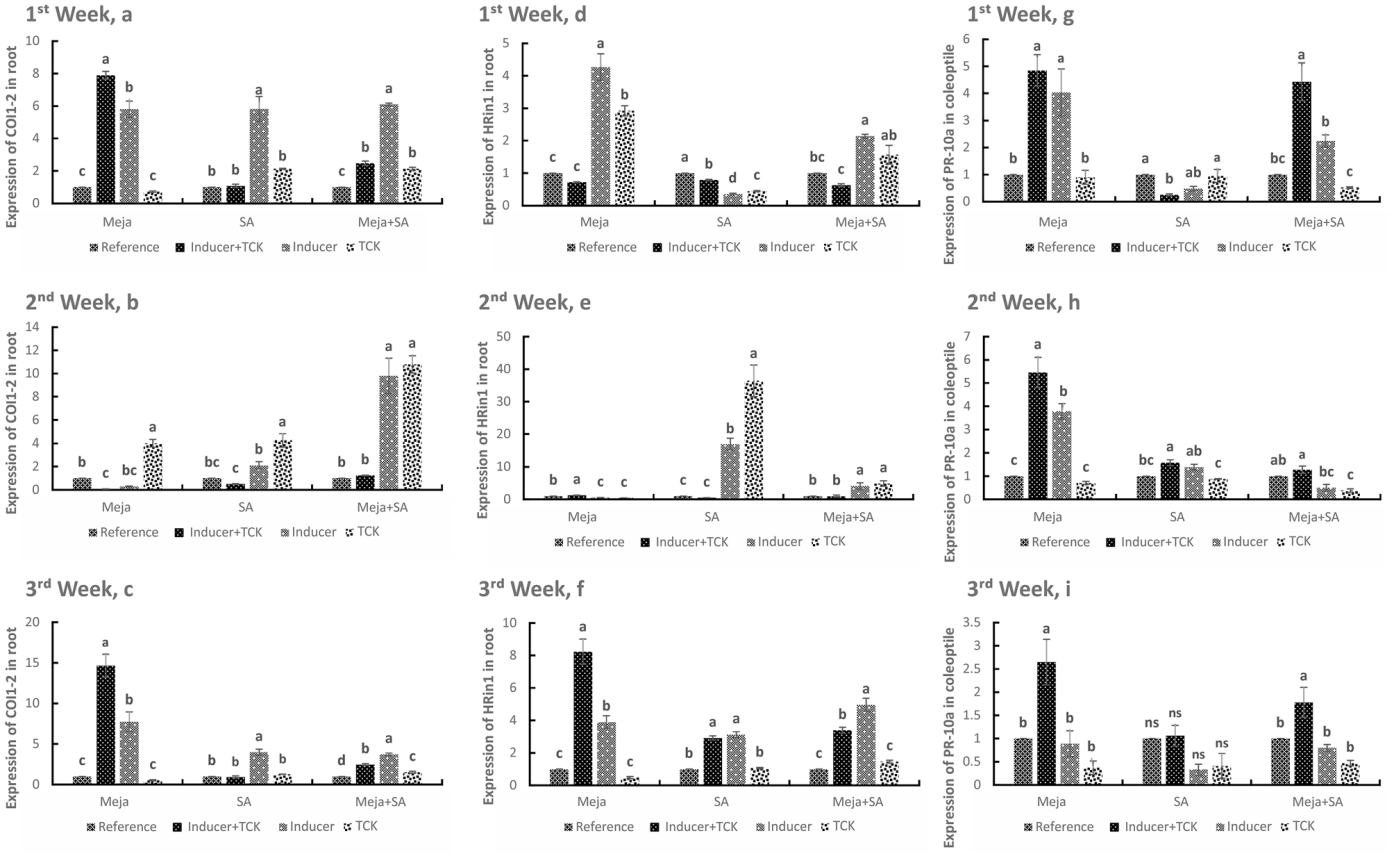
Time course of CoI1-2, HRin1 and PR-10a transcript expression in roots and coleoptiles in treated inoculated (inducer + TCK), treated non-inoculated (inducer), non-treated inoculated (TCK) plants. Plants receiving no inducer of resistance or TCK inoculation were used as controls. Treated mean application of inducers of resistance and inoculated mean application of TCK are shown. The treatments within the time interval followed by the same letters are not statistically significant using Tukey’s LSD test (*P* < 0.05).

COI1-2 levels were similarly upregulated by Meja, SA and Meja + SA after treatment for 1, 2 and 3 weeks in the inducer + TCK, inducer and TCK groups compared to the reference group (Fig. 2a–c). In the 1 week Meja treatment, the transcriptional levels of COI1-2 were 7.9-fold and 5.81-fold higher in the inducer + TCK and inducer plants, respectively, compared to reference. After 1 week, the COI1-2 levels were increased by 5.82-fold and 6.10-fold in the SA and Meja + SA treatments, respectively, in the inducer plants compared to reference (Fig. 2a). After 2 weeks of treatment, the highest transcriptional level of COI1-2 was in the Meja + SA treatment with compared increases of 1.24-fold, 9.8-fold and 10.79-fold in the inducer + TCK, inducer and TCK groups, respectively, compared to reference (Fig. 2b). However, the highest expression level of COI1-2 occurred after 3 weeks of treatment compared to treatments for 1 and 2 weeks. In the inducer + TCK and inducer groups after 3 weeks of Meja treatment, the COI1-2 levels increased by 14.65-fold and 7.74-fold, respectively, compared to the reference group (Fig. 2c). There was no statistically significant difference in COI1-2 expression in the inducer + TCK and TCK groups after SA application compared to the reference, but the COI1-2 expression level was 3.99-fold and 3.73-fold higher in the inducer groups after treatments with SA and Meja + SA, respectively, compared to the reference (Fig. 2c).

The HRin1 expression levels after treatment for 1, 2 and 3 weeks in the inducer + TCK, inducer, TCK and reference groups were also analysed (Fig. 2d–f). After treatment with Meja for 1 week, the HRin1 expression levels in the inducer + TCK, inducer and TCK groups were increased by 0.72-fold, 4.27-fold and 2.92-fold, respectively, compared to the reference. There was no statistically significant difference between the Meja + TCK group and the reference group. The HRin1 expression level after SA treatment decreased in the inducer + TCK and inducer groups compared to the reference. For the Meja + SA treatment, HRin1 expression levels were upregulated in the inducer treatment and TCK treatment (Fig. 2d). After SA treatment for 2 weeks, the expression of HRin1 increased by 17-fold in the inducer compared to the reference. Similarly, HRin1 expression was increased by 4.11-fold in the inducer treatment after Meja + SA application compared to the reference (Fig. 2e). After Meja treatment for 3 weeks, HRin1 expression was increased by 8.23-fold and 3.9-fold in the inducer + TCK and inducer groups, respectively, compared to the reference. After SA application for 3 weeks, HRin1 expression increased by 2.92-fold and 3.12-fold in the inducer + TCK and inducer treatments, respectively, compared to the reference. Moreover, after Meja + SA application for 3 weeks, HRin1 expression was increased by 3.39-fold and 4.96-fold in the inducer + TCK and inducer treatments, respectively, compared to the reference (Fig. 2f).

The PR-10a expression in coleoptiles after treatment with Meja and Meja + SA was generally upregulated in the inducer + TCK and inducer groups compared to the reference. After SA application, the expression of PR-10a was downregulated in the inducer + TCK group compared to the reference, and no significant difference was found in the TCK treatment compared to the reference (Fig. 2g). While Meja induced the highest PR-10a expression at 2 weeks, reaching increases of 5.46-fold and 3.78-fold in the inducer + TCK and inducer treatments, respectively, compared to the reference, the PR-10a expression in the TCK group was decreased by 0.71-fold compared to the reference. Moreover, a low level of PR-10a expression was detected after SA and Meja + SA treatments at 2 weeks (Fig. 2h). The PR-10a expression was high after treatment with Meja for 3 weeks in the inducer + TCK treated plants compared to the reference. There was no significant difference in the inducer and TCK groups compared to the reference group after treatment with Meja and Meja + SA. Additionally, no significant change in expression was observed in all groups after treatment for 3 weeks with SA and TCK compared to the control (Fig. 2i).

### COI1-1, COI1-2 and HRin1 transcriptional levels in coleoptiles after treatment of wheat plants with resistance inducers

**Figure 3:**
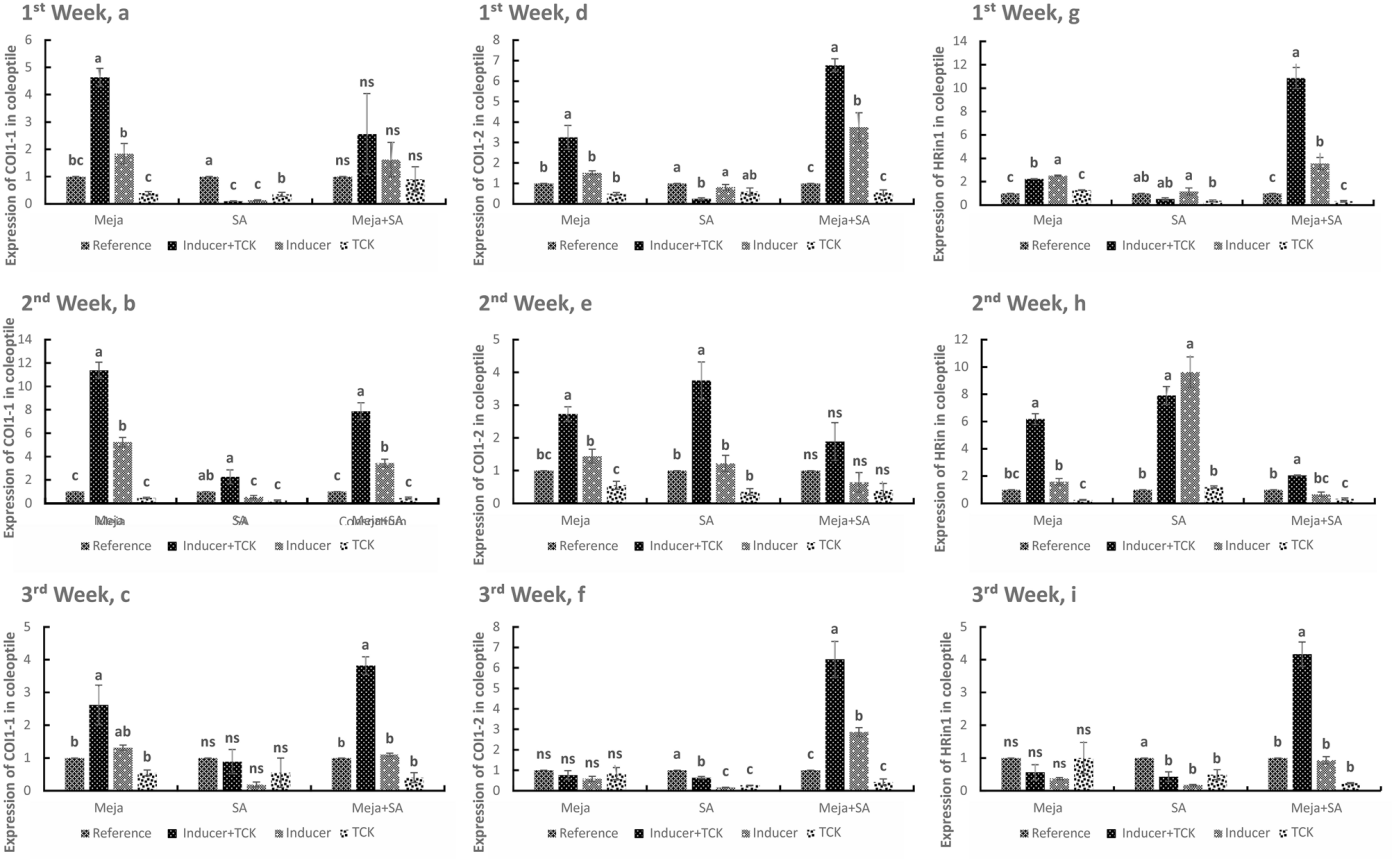
Time course of COI1-1, CoI1-2 and HRin1 transcript expression in coleoptiles in treated inoculated (inducer + TCK), treated non-inoculated (inducer), non-treated inoculated (TCK) plants. Treated mean application of inducers of resistance and inoculated mean application of TCK are shown. Plants receiving no inducer of resistance or TCK inoculation were used as controls. The treatments within the time interval followed by the same letters are not statistically significant using Tukey’s LSD test (*P* < 0.05).

COI1-1 expression showed a clear response to Meja in the inducer + TCK and inducer groups after 1 week with increases of 4.64-fold and 1.84-fold, respectively, compared to the reference. Compared to the reference, COI1-1 expression was downregulated after SA application for 1 week, but there was no significant change in COI1-1 expression after Meja + SA treatment for 1 week (Fig. 3a). Compared to the reference, Meja upregulated COI1-1 expression in the inducer + TCK and inducer groups by 11.39-fold and 5.24-fold, respectively, at 2 weeks (Fig. 3b). After 3 weeks of treatment with Meja and Meja + SA, COI1-1 expression was increased by 2.62-fold and 3.82-fold in the inducer + TCK treatments, respectively, compared to the reference, and there was no significant difference in SA application in the inducer + TCK and inducer treatments compared to the reference group (Fig. 3c).

COI1-2 expression in the coleoptile was upregulated by Meja and Meja + SA after treatment for 1 week in the inducer + TCK and inducer treatments compared to the reference (Fig. 3d). COI1-2 expression was increased by 3.25-fold after Meja treatment for 1 week in the in the inducer + TCK group compared to the reference. Similarly, COI1-2 expression was increased by 6.77-fold and 3.74-fold after treatment with Meja + SA for 1 week in the inducer + TCK and inducer groups, respectively, compared to the reference. With regard to SA application, there was no significant difference in COI1-2 expression when comparing the inducer group to the reference group. However, downregulated COI1-2 expression was observed in the inducer + TCK group compared to the reference (Fig. 3d). A low level of COI1-2 expression was noted for Meja and SA after 2 weeks in the inducer + TCK and inducer groups compared to the reference, and the expression level of COI1-2 was downregulated in the TCK group compared to the reference after treatment with Meja and SA. No statistically significant difference was present after treatment with Meja + SA for 2 weeks in all groups (Fig. 3e). However, downregulation of COI1-2 was observed after SA application for 3 weeks compared to the reference, and no statistically significant change in COI1-2 expression was observed after Meja treatment. However, COI1-2 expression reached a peak after treatment with Meja + SA for 3 weeks with increases of 6.42-fold and 2.87-fold in the inducer + TCK and inducer plants, respectively, compared to the reference (Fig. 3f).

HRin1 expression in the coleoptile was slightly upregulated by Meja treatment and significantly upregulated by the Meja + SA treatment but downregulated by SA treatment in the inducer + TCK and inducer groups. HRin1 expression in the inducer + TCK and inducer groups was increased by 2.23-fold and 2.51-fold, respectively after treatment with Meja compared to the reference. Treatment with Meja + SA in the inducer + TCK and inducer groups increased HRin1 expression by 10.86-fold and 3.55-fold, respectively, compared to reference (Fig. 3g). After treatment for 2 weeks, maximum relative expression of HRin1 was noted after treatment with SA compared to treatment with Meja and Meja + SA. SA treatment increased HRin1 expression by 7.91-fold and 9.62-fold in the inducer + TCK and inducer groups, respectively, compared to reference. Compared to reference, Meja treatment increased HRin1 expression by 6.18-fold in the inducer + TCK group, but treatment with Meja + SA increased HRin1 expression by only 2.05-fold (Fig. 3h). After 3 weeks, no statistically significant change in HRin1 expression was present after treatment with Meja treatment, but HRin1 expression was downregulated by SA treatment in all groups. However, HRin1 expression was increased by 4.17-fold after treatment with Meja + SA in the inducer + TCK group compared to the reference (Fig. 3i).

The above relative expression results showed that the inducers of resistance upregulated the expression levels of defence genes to protect against TCK after 1, 2 and 3 weeks. However, the expression changes were not consistent for specific periods of time. For example, Meja showed the best results at 1 week for one gene but not for all genes compared to SA and Meja + SA. Comparatively, inducers of resistance increased the expression level of defence genes in highly susceptible cultivars, suggesting that TCK, a bunt pathogen, can be controlled by the application of different inducers of resistance. More importantly, the overall expression levels in coleoptiles were low compared to roots, indicating that inducers of resistance degrade the fungal hyphae into roots and hyphae, thus preventing movement into the upper parts of plants.

### Hyphae development in wheat roots and coleoptiles in inoculated, non-inoculated and treated inoculated plants

**Figure 4:**
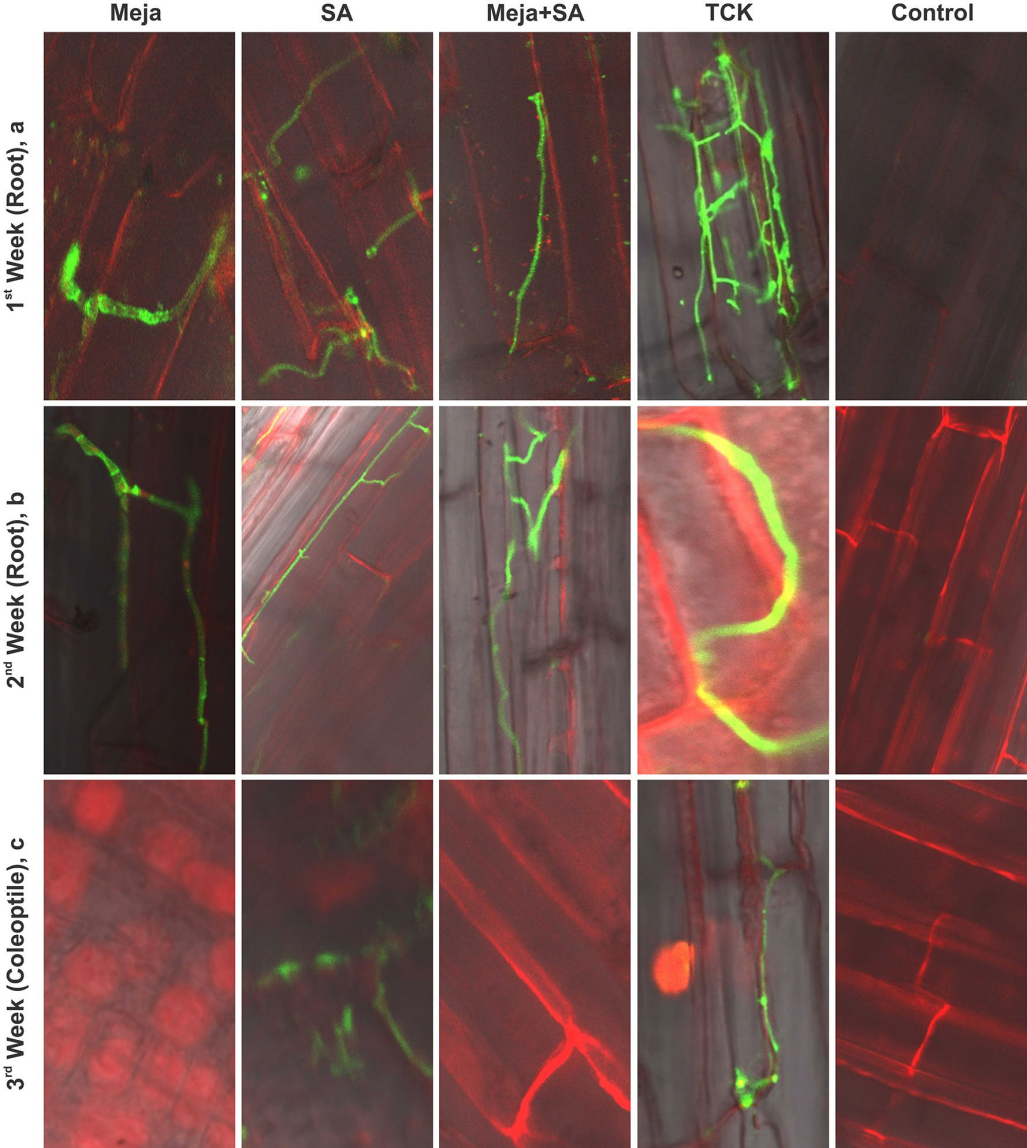
Infestation of TCK in wheat roots and coleoptiles as indicated by staining with WGA-AF 488 (for hyphae) and propidium iodide (for root cell). Plants receiving no inducer of resistance or TCK inoculation were used as controls. (**a**) At 1 week, hyphae excessively occupied rhizodermal and cortical cells of the roots. Presence of hyphae was visualized in treated inoculated (TCK + inducers), non-treated inoculated (TCK), and control plants. *(Meja)* Presence of hyphae in Meja-treated plants. Hyphae moved within the cortical cells. After penetrating the cortical cells, the hyphae moved into the endodermis and vascular bundles. *(SA)* Hyphae appeared in the epidermal cells of SA-treated roots. *(Meja* + *SA)* Hyphae were observed in the epidermal cells of Meja + SA-treated roots. *(TCK)* The epidermal structures were highly infested in the non-treated inoculated (TCK) plants compared to all other treated plants and controls. *(Control)* No hyphae were observed in the root structures of control plants. Scale bars = 50 µm, 50 µm, 50 µm, 25 µm and 75 µm for Meja, SA, Meja + SA, TCK and control, respectively. (**b**)* (Meja)* Presence of hyphae in Meja-treated plants. Hyphae moved within the cortical cells and penetrated the cortical cells. *(SA)* Long hyphae appeared on the cortical and sub-epidermal cells of SA-treated roots. *(Meja* + *SA)* Hypha were observed on cortical cells in Meja + SA-treated roots. *(TCK)* The sub-epidermal structures were highly infested in the non-treated inoculated (TCK) plants compared to all other treated and control plants. *(Control)* No hyphae were observed in the root structures of the control plants. Scale bars = 25 µm, 50 µm, 50 µm, 25 µm and 50 µm for Meja, SA, Meja + SA, TCK and control, respectively. *(c) (Meja)* Hyphae were not present in Meja-treated plants. *(SA)* In the SA treatment, small amounts of hyphae were observed. Hyphae did not wrap around the sub-epidermal cells properly. *(Meja* + *SA)* There was no presence of hyphae in Meja + SA-treated plants. *(TCK)* The cortical cells were ruptured in the non-treated inoculated plants, indicating that the hyphae moved from the roots to other upper parts of the inoculated control plants. *(Control)* No hyphae were observed into root structures in the mock plants. Scale bars = 50 µm, 25 µm, 50 µm, 25 µm and 50 µm for Meja, SA, Meja + SA, TCK and control, respectively.

To track the hyphae in roots, roots treated with different inducers of resistance were analysed by confocal microscopy. In general, we observed a gradual decrease of fungal colonization and propagation with time intervals (Fig. 4a–c). At germination, hyphae started from small tips, and they formed a hyphal network on and inside the roots. Hyphae moved into sub-epidermal cells through intercellular spaces where they branched and continued to grow. Sub-epidermal hyphae penetrated spaces between primary cell walls and plasma membranes of cortical cells. Hyphae penetrated epidermal cells and reached root cortical cells in the treated inoculated (TCK + inducers) and inoculated control (only TCK) plants, but there was no prevalence of hyphae into the non-inoculated plants. However, cortical cells were attacked more harshly in the inoculated control plants (Fig. 4a). These results showed that Meja, SA and their combination (Meja + SA) played pivotal roles in the control of *T. controversa* hyphae movement into the roots 1 week post-inoculation of the inducers of disease resistance. At 2 weeks, similar results were observed in all treated, inoculated control plants, but the hyphae were weaker than they were at 1 week (Fig. 4b). To confirm these observations, infected coleoptiles were stained with WGA-AF 488 at week 3 to diagnose the presence or movement of hyphae from roots to the upper parts of plants. The confocal results showed that there was no prevalence of hyphae movement into coleoptiles in the treated plants. Moreover, some broken hyphae were observed in coleoptiles after SA treatment, but fungal hyphae were present in the inoculated control (Fig. 4c). Therefore, we tracked *T. controversa* into different sub-cells of the anthers using confocal microscopy in inoculated control and treated inoculated plants.

### Hyphae proliferation into epidermal and sub-epidermal cells of anthers in non-treated inoculated plants

**Figure 5:**
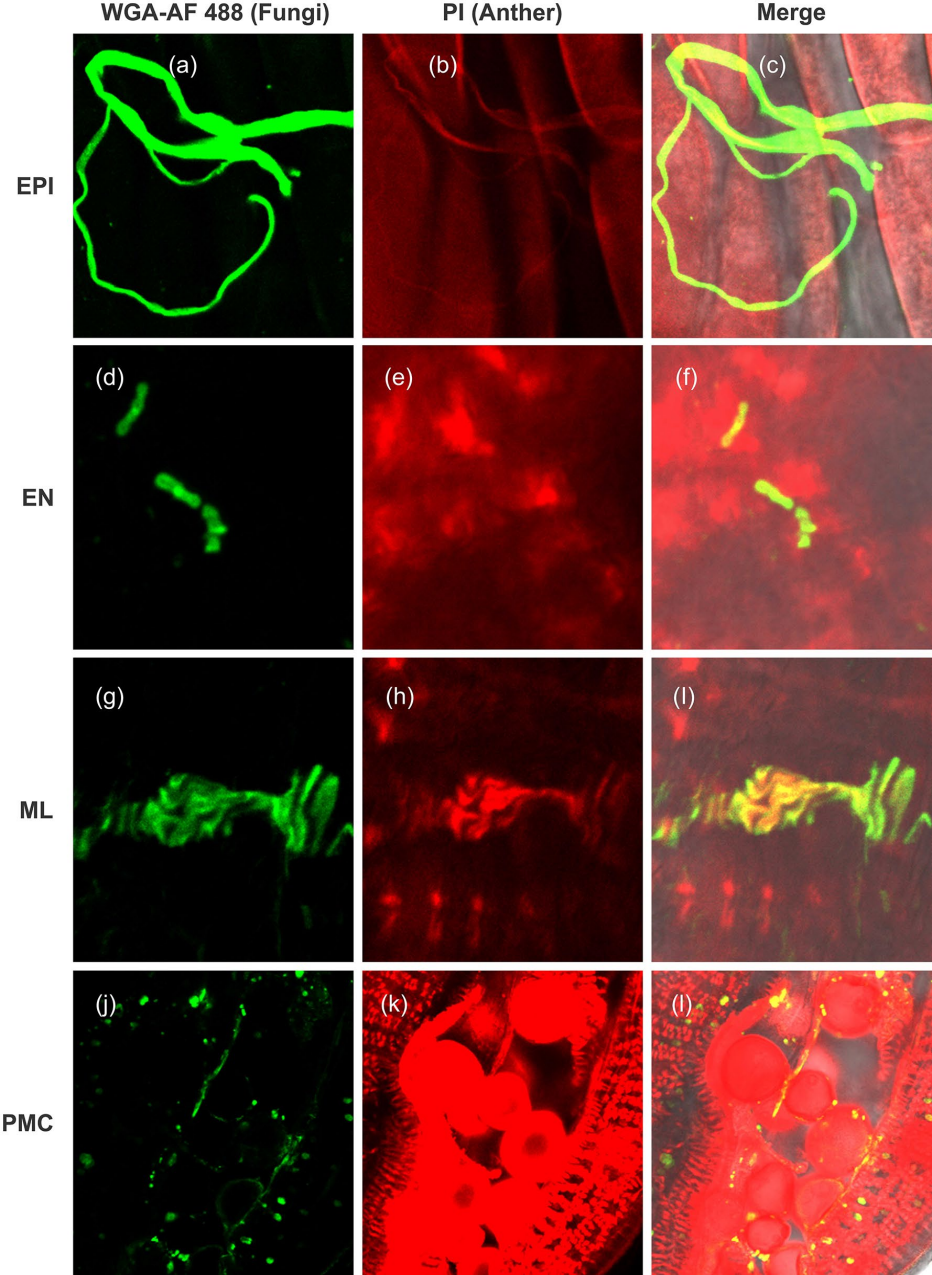
TCK is present on the epidermal and sub-epidermal cells of the anther. WGA-AF488 appeared green in live fungal tips, while PI appeared red colour in dead anther cells. (**a**–**c**) Hyphae were located on the EPI cells (scale bars = 25 µm). (**d**–**f**) Hyphae were located on the EN cells (scale bars = 7.5 µm). (**g**–**i**) Hyphae were located on the ML cells, scale bars = 10 µm. (**j**–**l**) Hyphae were located on the PMCs (scale bars = 75 µm).

To investigate the effect of *T. controversa* on anther cells, anthers were stained with WGA-AF 488 and PI to determine the establishment and proliferation of fungal hyphae in anther cells. Hyphae were observed on epidermal (EPI) and sub-epidermal cells, including the endothecium (EN), middle layer (ML) and pollen mother cells (PMCs) (Fig. 5a–l). Additionally, we investigated hyphal severity on EPI cells at different time intervals in non-treated inoculated plants. The results showed that at 6- and 7-days post inoculation (dpi), hyphae were observed on anther EPI cells (Fig. S1a–f and Table 1). The intensity of the hyphae increased with time, and the maximum fungal intensity on EPI cells was recorded at 8, 9 and 10 dpi (Fig. S1g–o and Table 1).

**Table 1:** Infection of TCK on the epidermal and sub-epidermal cells of the anthers at different days post inoculation (DPI).

DPI	Fungal location
6	EPI
7	EPI
8	EPI, EN
9	EPI, EN, ML
10	EPI, EN, ML, PMC

Epidermal cell (EPI), Endothecium cell (EN), Middle layer cell (ML), Pollen mother cell (PMC).

### Inducers of resistance eliminate *T. controversa* from anther cells

**Figure 6:**
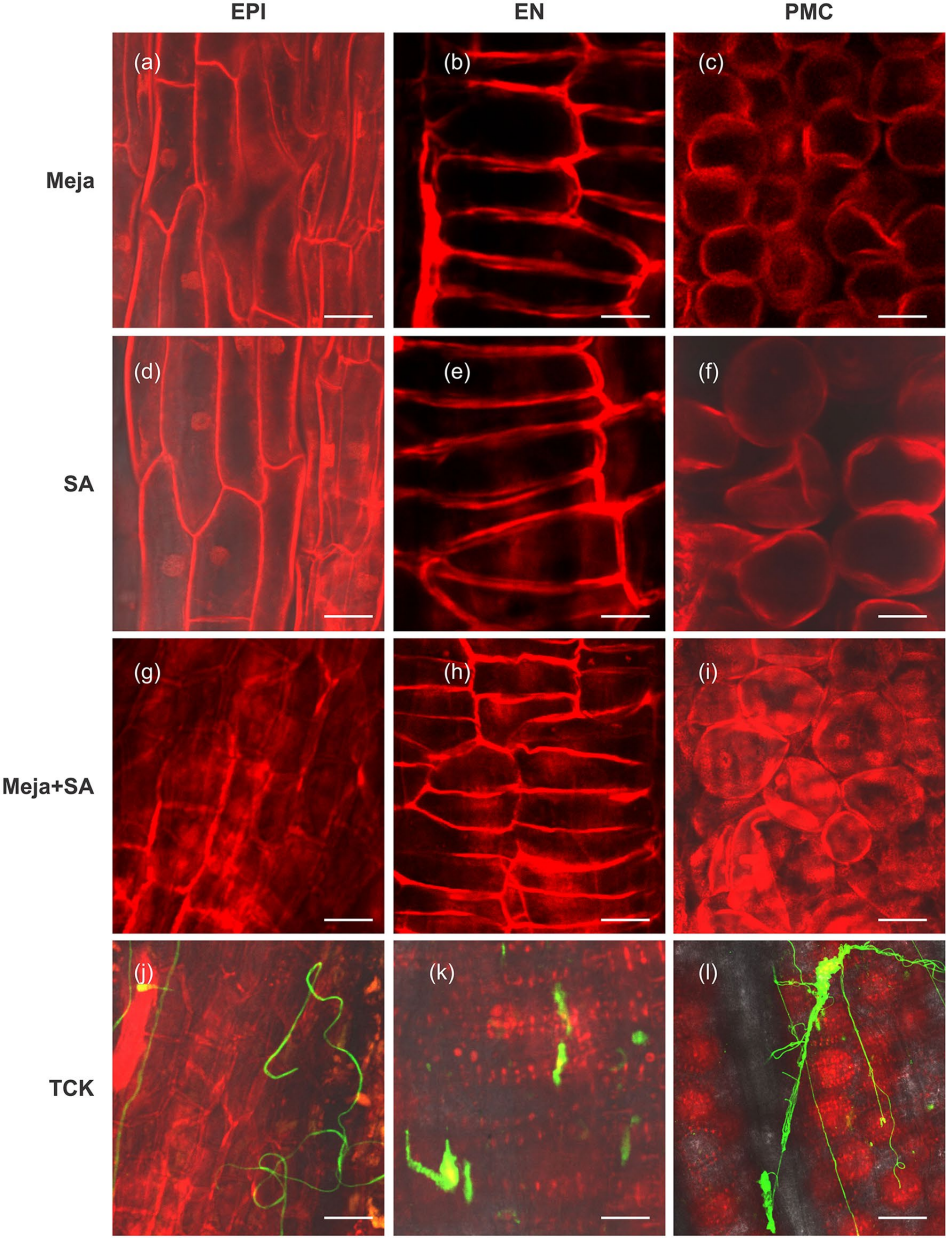
TCK is present on the epidermal and sub-epidermal cells of the anthers in non-treated inoculated (TCK) and treated inoculated (inducers + TCK) plants compared to control plants. WGA-AF488 appeared green in hyphae, while PI appeared red in dead anther cells. (**a**–**c**) There were no hyphae on the EPI, EN and PMCs of anthers in Meja-treated plants (scale bars = 50 µm, 25 µm and 100 µm, respectively). (**d**–**f**) There were no hyphae on the EPI, EN and PMCs of anthers in SA-treated plants (scale bars = 50 µm, 25 µm and 50 µm). (**g**–**i**) Similarly, we did not observe hyphae on the EPI, EN and PMCs in Meja + SA-treated plants (scale bars = 25 µm, 50 µm and 50 µm, respectively). (**j**–**l**) Hyphae were located on the EPI, EN and PMCs in non-treated inoculated (TCK) anthers (scale bar = 25 µm, 25 µm and 50 µm, respectively.

To track the hyphae of *T. controversa* in treated inoculated and inoculated control plants, fungal penetration into the EPI, EN and PMCs (anther cells) was analysed by confocal laser scanning microscopy (Table 1). No hyphae was observed in these three cell types (Fig. 6a–i), but highly infestation of hyphae was observed in the inoculated control anthers (Fig. 6j–l). These results indicated that there was no *T. controversa* hyphae penetration into the EPI, EN and PMCs of anthers after treatment with Meja (Fig. 6a–c). Similarly, in SA and Meja + Sa treatments, no *T. controversa* hyphae penetration into the EPI, EN, and PMCs was observed (Fig. 6d–i). However, in inoculated control anthers, hyphae encased the EPI, EN and PMCs, resulting in cell shrinkage and rupture (Fig. 6j–l). Additionally, the physical and morphological structures of the EPI, EN and PMCs after treatment with Meja, SA and Meja + Sa indicated healthy cells compared to inoculated control anthers. These findings revealed that inducers of resistance played pivotal roles in inhibiting the penetration of *T. controversa* into epidermal and sub-epidermal cells of the highly susceptible cultivar of wheat.

### Inducers of resistance enhance wheat resistance to *T. controversa*

**Figure 7:**
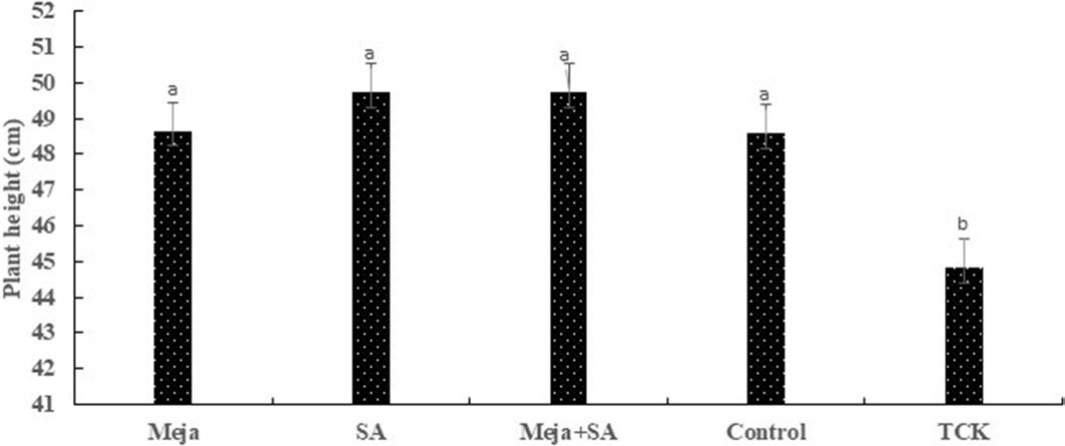
Plant height of the treated inoculated, TCK-inoculated and control plants was measured and analysed using a LSD test (Statistix 8.1 software). Twelve replicates were used for comparing the plant height of treated inoculated, control and TCK-inoculated plants. Different letters represent the statistical significance among the different treatments.

To further investigate the diseased symptoms in treated inoculated, non-treated inoculated and control plants, we observed three seeds from spikes of each treatment. The results TCK hyphae were not present in the seeds from the treated inoculated and control plants (Fig. S2a–d), while clear and obvious bunted seeds were found in the non-treated inoculated plants (Fig. S2e). These data suggested that inducers of resistance contribute to control the dwarf bunt disease of wheat. Furthermore, we compared the treated inoculated, non-treated inoculated and control seeds collected from the spike of every treatment to investigate the symptoms in more detail. Normal seeds were observed in treated inoculated and control plants (Fig. S3a,c,e,g), while bunted seeds with clear symptoms were observed in non-treated inoculated plants (Fig. S3b,d,f,h). Additionally, we cut the seed longitudinally to compared the black powder inside the seeds in the non-treated inoculated plants to the treated inoculated and control plants. There were millions of teliospores in the seeds collected from non-treated inoculated spikes (Fig. S4b,d,f,h), while a white-like powder was observed in the treated inoculated and control seeds (Fig. S4a,c,e,g). Plant height is parameter affected by TCK, and stunted growth was observed in the infected fields, indicating that TCK-infected plants showed stunted growth compared to treated inoculated and control treatments (Fig. 7).

## Discussion

Wheat is one of major staple food crops worldwide. However, yield losses of wheat due to *T. controversa* under favourable conditions can reach up to 70–80% in some wheat growing areas. Controlling dwarf bunt disease of wheat by resistant cultivars is a laborious and time-consuming process. Here, we reported the role of inducers of resistance to control dwarf bunt in the roots and coleoptiles, and we analysed the infection mechanism of TCK in anther epidermis and sub-epidermal cells. Assays using confocal microscopy, qRT-PCR and physiological traits showed that inducers of resistance (i.e., Meja, SA and combination of Meja + SA) enhanced resistance to dwarf bunt disease in a highly susceptible wheat cultivar. Overall, the data demonstrated that inducers of resistance are important mediators that increase the defence mechanism of wheat against *T. controversa*. New environmentally friendly molecules to protect against wheat biotic stress should be investigated to broaden insights on abiotic and biotic stress signalling pathways in different plant species. In this experiment, exogenous application of Meja, SA and Meja + SA was used to control the dwarf bunt pathogen in the roots and coleoptiles by activating different root defence genes. SA is a defence signal molecule associated with resistance to hemibiotrophic and biotrophic pathogens^3,32,33^. Many defence-related genes of plants are associated with the SA pathway^18,34^. Our results showed that the expression of defence genes in roots and coleoptiles of SA- and Meja- treated plants was higher compared to control or non-treated plants (Figs. 1, 2, 3). Additionally, our results revealed that the invasion capability of *T. controversa* decreased with the passage of time after treatment with SA, Meja and Meja + SA (Fig. 4). Thus, we concluded that inducers of resistance significantly control TCK in the roots and coleoptiles. Movement of the hyphae into roots and into vegetative and reproductive parts of the plants almost disappeared after treatment for 3 weeks with inducers of resistance.

Pathogenesis-related (PR) proteins play key roles in the defence mechanism of plants against various pathogen infections^35,36^. PR proteins are a group of functionally diverse inducible proteins that accumulate in plant tissue in response to pathogen infection^37^. PR proteins directly threaten pathogen integrity or release biochemicals by their enzymatic activity as elicitor molecules, which activates other plant defence-related pathways^38^. Most PR proteins play a role in fungal/oomycete pathogens. PR-10a and HRin1 are associated with the defence response in cereals, and they help plants to overcome the infection of pathogens^39,40^. PR-10a-like proteins have been described in the root epidermis and root hair cells of pea plants^41^. HRin1 activates the hypersensitive response in plants, which prevents the establishment of the pathogens^31^. Catalase genes are the major component of oxidative stress metabolism^27,28^. Catalases, along with other scavenging enzymes for reactive oxygen species (ROS), such as superoxide dismutase (SOD) and ascorbate peroxidase, are of fundamental importance to the survival of organisms undergoing oxidative and pathogenic stress^42^. Previous studies have shown that the expression of COI1-1 and COI1-2 genes enhances resistance to soilborne pathogens in various crops^30^. Meja signalling is mediated by COI1-1 and COI1-2 genes, which are F-box components of the Skp1-Cullin F-box protein (SCF ^COI1^) ubiquitin E3 ligase^43^. COI is also involved in apical dominance^44^, leaf senescence^45^, ethylene-induced root growth inhibition in the light in *Arabidopsis thaliana*^46^ and inositol polyphosphates^47^. Therefore, we applied the inducers of resistance to the highly susceptible wheat cultivar to activate the defence genes in the roots and coleoptiles to reduce the level of dwarf bunt pathogen. The roots and coleoptiles of wheat seedlings exhibited changes in gene expression during *T. controversa* inoculation and treatment with the inducers of resistance. The expression of defence-related gene expression of inoculated and treated plants was higher than the control plants (Figs. 1, 2, 3).

In this study, we also analysed the effect of *T. controversa* on anther development to investigate whether *T. controversa* present on epidermal and sub-epidermal cells of the anther and its intensity level increase on anther cells with the passage of time. TCK severity increased over time, resulting in cell wall rupturing in plants (Figs. 5, S1). Infection of young anthers prior to cell-fate differentiation results in total disruption of anther internal lobe development because all sub-epidermal cells are abnormal and cannot produce differentiated features of the EN, ML and PMCs^48^. Fungal penetration into roots is a gradual progress. Spores start to germinate on the soil surface, enter into roots, move to nodes and finally reach into anthers. We first observed fungal hyphae only on EPI cells, but over time, the fungal hyphae appeared on the EN, ML and PMCs (Fig. 5). These results indicated that establishment of hyphae on cells and sub-cells destroys the normal process of cell expansion and division. Additionally, the intensity level of the hyphae increased as the anther increased in size and matured (Fig. S1). Healthy anthers produced healthy ovaries and grains, while infected anthers produced bunted ovaries. Finally, ovaries and grains produced by the infected anthers turned into a black coloured powder containing teliospores (Figs. S2, S3, S4).

Our finding supported the hypothesis that inducers of resistance activate the defence responses in the roots and coleoptiles and that they eliminate the pathogen movement to the vegetative and reproductive parts of the plants. Exogenous application of inducers of resistance was effective in reducing the infection of *T. controversa* into roots, coleoptiles and anther tissues. Our results revealed that treatment with the inducers of resistance decreased the intensity level of the *T. controversa* hyphae at 3 weeks with no obvious systems observed at any stage of the plants and no teliospores in the grains. In contrast, clear hyphae were observed on the epidermis and sub-epidermal cells of the anthers in non-treated inoculated plants, and intensity level of hyphae increased with the passage of time as the anthers matured. Additionally, plant height was reduced by *T. controversa* but increased by the inducers of resistance.

## Materials and methods

### Plant material, fungal isolate and treatments

Wheat (*Triticum aestivum* L.) cv. Dongxuan 3 (highly susceptible) seeds were obtained from the Institute of Plant Protection, Chinese Academy of Agricultural Sciences, China. The TCK strain was kindly provided by Blair Goates from United States Department of Agriculture (USDA), Agriculture Research Service.

The Dongxuan 3 specimens (30-day seedlings) were grown in a greenhouse incubator (Percival, America) under a 24 h light (6 ± 2 °C and 70% relative humidity) regime. The wheat seedlings were inoculated with TCK according to the method of our laboratory. Briefly, TCK was grown on 2% agar media and incubated for 60 days at 5 °C under 24 h light. The TCK mycelia were harvested in a laminar flow hood by adding 5 ml of ddH_2_O into each TCK culture plate. TCK hyphae were injected 5 times into the root zone at 2-day intervals. Hyphae were also injected into spikes during ear emergence from boot, corresponding to Zadok’s growth stages 49, 50 and 51^49^. The TCK-inoculated plants were kept in an incubator at 6 ± 2 °C until they were treated with the inducers of resistance.

The inducers of resistance were applied 6 and 8 days after TCK inoculation into the wheat root zones and seedlings as previously reported^17^. Roots and seedlings were sprayed with 20 mM SA (APE × BIO, Catalogue No. B1092) and 100 µM Meja (APE × BIO), and Tween 20 (0.01%) was added as a surfactant to the SA and Meja treatments. The inducers of resistance were applied to both non-inoculated and TCK-inoculated plants. Plants receiving only TCK were considered as the non-treated inoculated plants, and plants receiving no inducer or TCK were considered as the control plants. Control plants were sprayed with Tween 20/water plus 0.1% ethanol.

### RNA extraction and cDNA synthesis

The roots and coleoptiles were harvested, immediately frozen in liquid nitrogen and ground in a 2-µL centrifuge tube by adding 5-mm beads. Total RNA was extracted from 0.8 g of root and coleoptile powder using the Solarbio Life Sciences kit (Beijing, China) according to the manufacturer’s instructions. The quantity and quality of RNA were analysed by a NanoDrop device (Denovix, Spectrophotometer, USA). First-strand cDNA was synthesized using 1 µg of purified total RNA, RT/RI enzyme and oligo (dT)_18_ primer (TransGen) following the kit’s instructions (TransGen). The synthesized cDNA was diluted 4 times in ddH_2_O to a final 80 µL volume, and 2 µL of the diluted cDNA was used as the template for real-time PCR analysis.

### Real-time quantitative RT-PCR (RT-qPCR) analysis of defence-related genes

RT-qPCR was performed using Top Green qPCR SuperMix (TransGen) in a volume of 20 µL according to the manufacturer’s instructions and applied to the QuantStudio 5 real-time PCR system (Applied Biosystems, Beijing, China) for quantification. Amplification of the wheat GPDH43 gene was used as an internal control for normalizing all data. We used the following RT-qPCR protocol: pre-denaturation at 95 °C for 10 min; and 40 cycles of 95 °C for 15 s, 58 °C for 30 s and 72 °C for 30 s. The 2^−ΔΔCt^ method^50^ was used to evaluate the relative expression of defence-related genes with three biological replicates and four technical replicates.

### Staining TCK in root and coleoptile tissues

Hyphae in root and coleoptile segments were stained with the wheat germ agglutinin-Alexa Flour 488 conjugate (WGA-AF 488) chitin-specific dye (Molecular probes/Invitrogen, P1304MP, Life Technologies, America), and plant cells were stained with propidium iodide (PI). Depending on the experiment, roots and coleoptiles were dipped in absolute ethanol for 30 min. Absolute ethanol was added three times after 35 min until the tissue turned white. Subsequently, both segments were incubated at room temperature for 5 min in 1 × phosphate-buffered saline (PBS; pH 7.4) containing each respective dye at 10 µg/ml. This process was repeated three times for optimal washing. PI (W11261, Life Technologies, USA) was used as a counterstain by adding it to the WGA-AF 488 staining solution at a final concentration of 10 µg/ml. Each segment of root and coleoptile was mounted on a glass slide for observation using a confocal microscope (TCS SP8, Leica, Germany). WGA-AF 488 was excited at a wavelength of 448 nm and detected at 510–550 nm.

PI was excited at a wavelength of 561 nm and detected at 570–730 nm. For every treatment, 15 to 20 replicates were assessed.

### TCK staining in anther tissue

Spikelets of non-treated inoculated plants were separated from spikes to recover the anthers, and all anthers from every dissected floret were incubated in absolute ethanol (95% ethanol) for 35 min. The protocols for staining and observing the fungal hyphae in anthers under confocal microscopy were same as those mentioned above. Moreover, 12 plants were selected for plant height comparison in all treatments, and they were measured using a ruler. The spikes were removed from the treated inoculated, non-treated inoculated and control plants for symptom comparison.

### Primers

All qRT-PCR primers are listed in Supplementary data (Table S1).

### Statistical analysis

Data were statistically analysed using one-way ANOVA followed by LSD test using SPSS software version 20.0. The mean and standard error were calculated using data from three independent biological samples and four technical replicates.

## Supplementary information

Supplementary Information.

## Abbreviations

TCK: *Tilletia controversa* Kühn
PI: Propidium iodide
WGA-AF488: Wheat germ agglutinin, Alexa Flour 488 conjugate
Meja: Methyljasmonate
SA: Salicylic acid

## References

[1] Shiferaw B (2013). Crops that feed the world 10. Past successes and future challenges to the role played by wheat in global food security. Food Secur..

[2] Wei X (2017). TaPIMP2, a pathogen-induced MYB protein in wheat, contributes to host resistance to common root rot caused by Bipolaris sorokiniana. Sci. Rep..

[3] Pieterse CMJ, Leon-Reyes A, Van der Ent S, Van Wees SCM (2009). Networking by small-molecule hormones in plant immunity. Nat. Chem. Biol..

[4] Zhang Z (2012). An R2R3 MYB transcription factor in wheat, Ta PIMP 1, mediates host resistance to Bipolaris sorokiniana and drought stresses through regulation of defense-and stress-related genes. New Phytol..

[5] Mathre DE (1996). Dwarf bunt: Politics, identification, and biology. Annu. Rev. Phytopathol..

[6] Gao L (2014). Development of a SCAR marker for molecular detection and diagnosis of *Tilletia controversa* Kühn, the causal fungus of wheat dwarf bunt. World J. Microbiol. Biotechnol..

[7] Muhae-Ud-Din G, Chen D, Liu T, Chen W, Gao L (2020). Characterization of the wheat cultivars against *Tilletia controversa* Kühn, causal agent of wheat dwarf bunt. Sci. Rep..

[8] Goates BJ (2012). Identification of new pathogenic races of common bunt and dwarf bunt fungi, and evaluation of known races using an expanded set of differential wheat lines. Plant Dis..

[9] Hoffman JA (1982). Bunt of wheat. Plant Dis..

[10] Purdy LH, Meiners JP, Hoffmann JA, Stewart VR (1963). Time of year of infection of winter wheat by dwarf bunt fungus. Phytopathology.

[11] Zouhar M, MaZákoVá J, ProkiNoVá E, VáňoVá M, Ryšánek P (2010). Quantification of *Tilletia caries* and *Tilletia controversa* mycelium in wheat apical meristem by real-time PCR. Plant Prot. Sci..

[12] Gisi U, Chet I, Gullino ML (2009). Recent Developments in Management of Plant Diseases.

[13] Mishra AK, Sharma K, Misra RS (2012). Elicitor recognition, signal transduction and induced resistance in plants. J. Plant Interact..

[14] Mengiste T, Chen X, Salmeron J, Dietrich R (2003). The BOTRYTIS SUSCEPTIBLE1 gene encodes an R2R3MYB transcription factor protein that is required for biotic and abiotic stress responses in Arabidopsis. Plant Cell.

[15] Yi SY (2004). The pepper transcription factor CaPF1 confers pathogen and freezing tolerance in Arabidopsis. Plant Physiol..

[16] Jones JDG, Dangl JL (2006). The plant immune system. Nature.

[17] Lu Z-X (2005). Inducers of resistance reduce common bunt infection in wheat seedlings while differentially regulating defence-gene expression. Physiol. Mol. Plant Pathol..

[18] Fujita M (2006). Crosstalk between abiotic and biotic stress responses: A current view from the points of convergence in the stress signaling networks. Curr. Opin. Plant Biol..

[19] Vlot AC, Dempsey DA, Klessig DF (2009). Salicylic acid, a multifaceted hormone to combat disease. Annu. Rev. Phytopathol..

[20] Glazebrook J (2005). Contrasting mechanisms of defense against biotrophic and necrotrophic pathogens. Annu. Rev. Phytopathol..

[21] Browse J (2009). Jasmonate passes muster: A receptor and targets for the defense hormone. Annu. Rev. Plant Biol..

[22] Veronese P (2003). In defense against pathogens. Both plant sentinels and foot soldiers need to know the enemy. Plant Physiol..

[23] Kreps JA (2002). Transcriptome changes for Arabidopsis in response to salt, osmotic, and cold stress. Plant Physiol..

[24] Uknes S (1992). Acquired resistance in Arabidopsis. Plant Cell.

[25] Ryals JA (1996). Systemic acquired resistance. Plant Cell.

[26] Schweizer P (1997). Jasmonate-inducible genes are activated in rice by pathogen attack without a concomitant increase in endogenous jasmonic acid levels. Plant Physiol..

[27] Navrot N, Rouhier N, Gelhaye E, Jacquot J (2007). Reactive oxygen species generation and antioxidant systems in plant mitochondria. Physiol. Plant..

[28] Quan L, Zhang B, Shi W, Li H (2008). Hydrogen peroxide in plants: A versatile molecule of the reactive oxygen species network. J. Integr. Plant Biol..

[29] Bai J (2018). Genome-wide identification and analysis of the COI gene family in wheat (*Triticum aestivum* L.). BMC Genom..

[30] Vijayan P, Shockey J, Lévesque CA, Cook RJ (1998). A role for jasmonate in pathogen defense of Arabidopsis. Proc. Natl. Acad. Sci..

[31] Okubara PA, Call DR, Kwak Y, Skinner DZ (2010). Induction of defense gene homologues in wheat roots during interactions with Pseudomonas fluorescens. Biol. Control.

[32] Adie BAT (2007). ABA is an essential signal for plant resistance to pathogens affecting JA biosynthesis and the activation of defenses in Arabidopsis. Plant Cell.

[33] Kawano T, Furuichi T (2007). Salicylic acid as a defense-related plant hormone. Salicylic acid: A plant hormone.

[34] Fan J, Hill L, Crooks C, Doerner P, Lamb C (2009). Abscisic acid has a key role in modulating diverse plant-pathogen interactions. Plant Physiol..

[35] Balasubramanian V, Vashisht D, Cletus J, Sakthivel N (2012). Plant β-1, 3-glucanases: Their biological functions and transgenic expression against phytopathogenic fungi. Biotechnol. Lett..

[36] Ameye M (2015). Priming of wheat with the green leaf volatile Z-3-hexenyl acetate enhances defense against *Fusarium graminearum* but boosts deoxynivalenol production. Plant Physiol..

[37] van Loon LC, Rep M, Pieterse CMJ (2006). Significance of inducible defense-related proteins in infected plants. Annu. Rev. Phytopathol..

[38] Edreva A (2005). Pathogenesis-related proteins: Research progress in the last 15 years. Gen. Appl. Plant Physiol..

[39] Rostoks N, Schmierer D, Kudrna D, Kleinhofs A (2003). Barley putative hypersensitive induced reaction genes: Genetic mapping, sequence analyses and differential expression in disease lesion mimic mutants. Theor. Appl. Genet..

[40] Miché L, Battistoni F, Gemmer S, Belghazi M, Reinhold-Hurek B (2006). Upregulation of jasmonate-inducible defense proteins and differential colonization of roots of Oryza sativa cultivars with the endophyte Azoarcus sp. Mol. Plant Microbe Interact..

[41] Mylona P (1994). The root epidermis-specific pea gene RH2 is homologous to a pathogenesis-related gene. Plant Mol. Biol..

[42] Blackman LM, Hardham AR (2008). Regulation of catalase activity and gene expression during Phytophthora nicotianae development and infection of tobacco. Mol. Plant Pathol..

[43] Yan J (2009). The arabidopsis CORONATINE INSENSITIVE1 protein is a jasmonate receptor. Plant Cell.

[44] Kim J (2013). New clothes for the jasmonic acid receptor COI1: Delayed abscission, meristem arrest and apical dominance. PLoS One.

[45] Shan X (2011). The role of Arabidopsis rubisco activase in jasmonate-induced leaf senescence. Plant Physiol..

[46] Adams E, Turner J (2010). COI1, a jasmonate receptor, is involved in ethylene-induced inhibition of Arabidopsis root growth in the light. J. Exp. Bot..

[47] Mosblech A, Thurow C, Gatz C, Feussner I, Heilmann I (2011). Jasmonic acid perception by COI1 involves inositol polyphosphates in *Arabidopsis thaliana*. Plant J..

[48] Gao L, Kelliher T, Nguyen L, Walbot V (2013). Ustilago maydis reprograms cell proliferation in maize anthers. Plant J..

[49] Tottman DR, Makepeace RJ, Broad H (1979). An explanation of the decimal code for the growth stages of cereals, with illustrations. Ann. Appl. Biol..

[50] Pfaffl MW (2001). A new mathematical model for relative quantification in real-time RT–PCR. Nucleic Acids Res..

