# Supplementary material for: Methyljasmonate and salicylic acid contribute to the control of Tilletia controversa Kühn, causal agent of wheat dwarf bunt
Source: Sci Rep. 2020 Nov 5;10:19175. doi: 10.1038/s41598-020-76210-2 (PMC7645591; doi:10.1038/s41598-020-76210-2)
Supplement: Supplementary file 1 — Supplementary Information. [file 41598_2020_76210_MOESM1_ESM.docx]

Title page for Supplementary Information

**Methyljasmonate and salicylic acid contribute to the control of *Tilletia controversa* Kühn, causal agent of wheat dwarf bunt**

**Ghulam Muhae-Ud-Din^1^, Delai Chen^1.2^, Taiguo Liu^1^, Wanquan Chen^1^, and Li Gao*^1^**

^1^State Key Laboratory for Biology of Plant Disease and Insect Pests, Institute of Plant Protection, Chinese Academy of Agricultural Sciences, Beijing 100193, P. R. China

^2^College of Plant Protection, Gansu Agricultural University, Lanzhou, Gansu Province 730070, P.R. China

*Correspondence: **Li Gao (**[xiaogaosx@hotmail.com](mailto:lgao@ippcaas.cn))


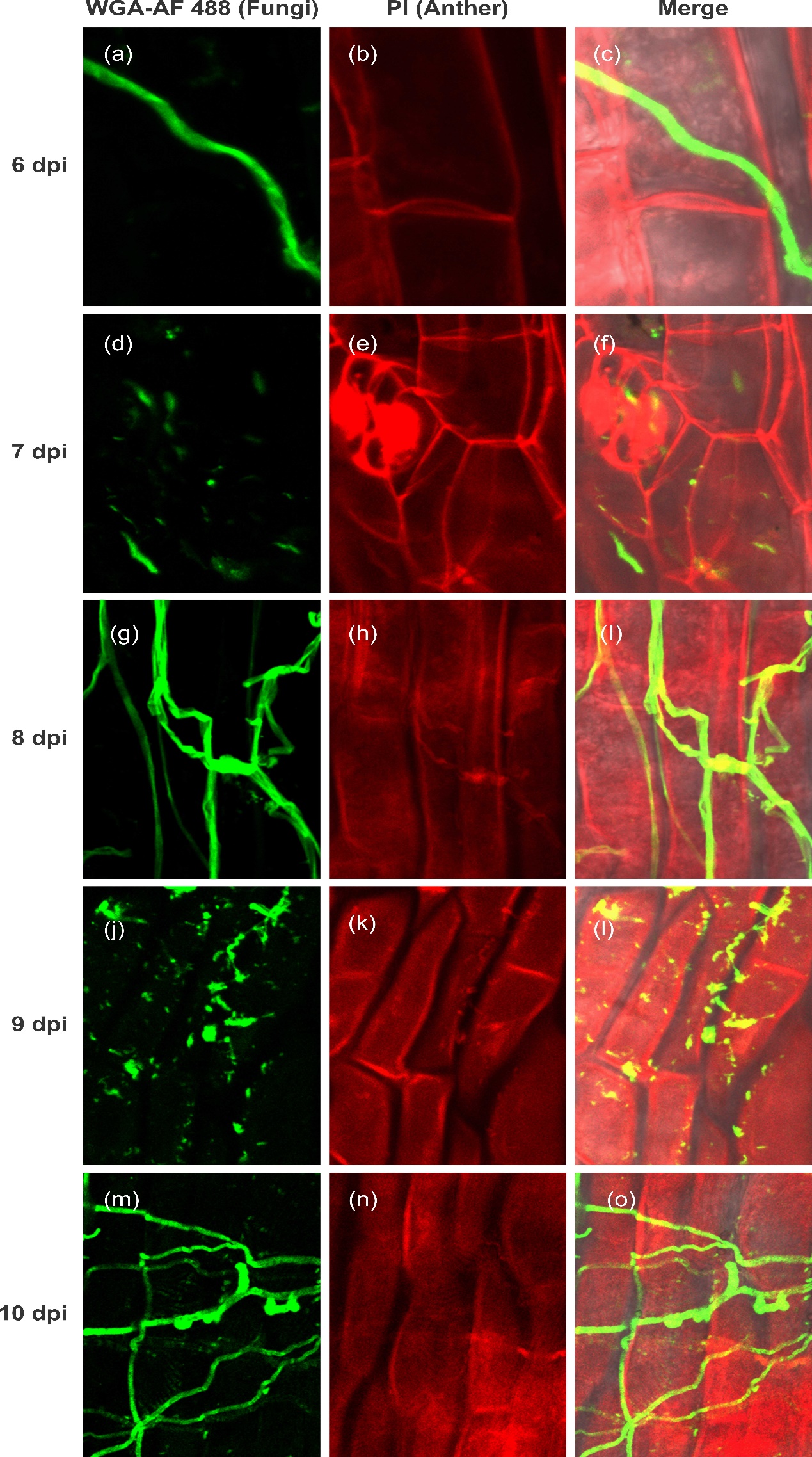


**Figure S1** Effect of TCK on EPI cells in different interval of times. (a-c) TCK after 6 dpi, scale bar = 7.5 µm. (d-f) TCK after 7 dpi, scale bar = 10 µm. (g-i) TCK after 8 dpi, scale bar, 25 µm. (j-k) TCK after 9 dpi, scale bar = 10 µm (m-o) TCK after 10 dpi, scale bar = 25 µm.


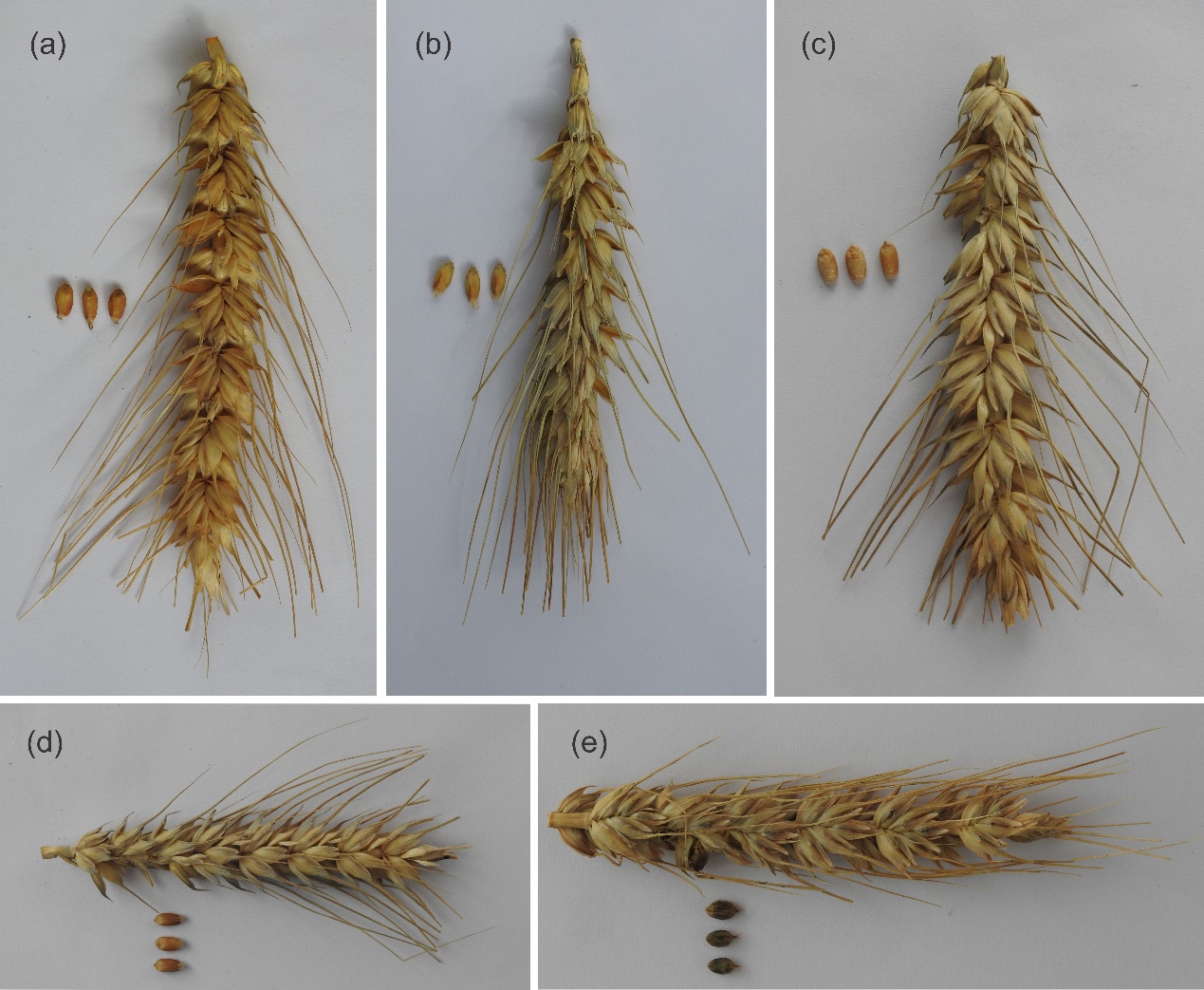


**Figure S2**. Compared spikes of treated inoculated, non-treated inoculated and control plants for disease symptoms. Three seeds removed from every spike and observed for disease symptoms. (a) Spike in Meja treatment plants. (b*)* Spike in SA treated plants. (c) Spike in Meja + SA treated plants. (d) Spike in control plants. (e) Spike in non-treated inoculated plants.


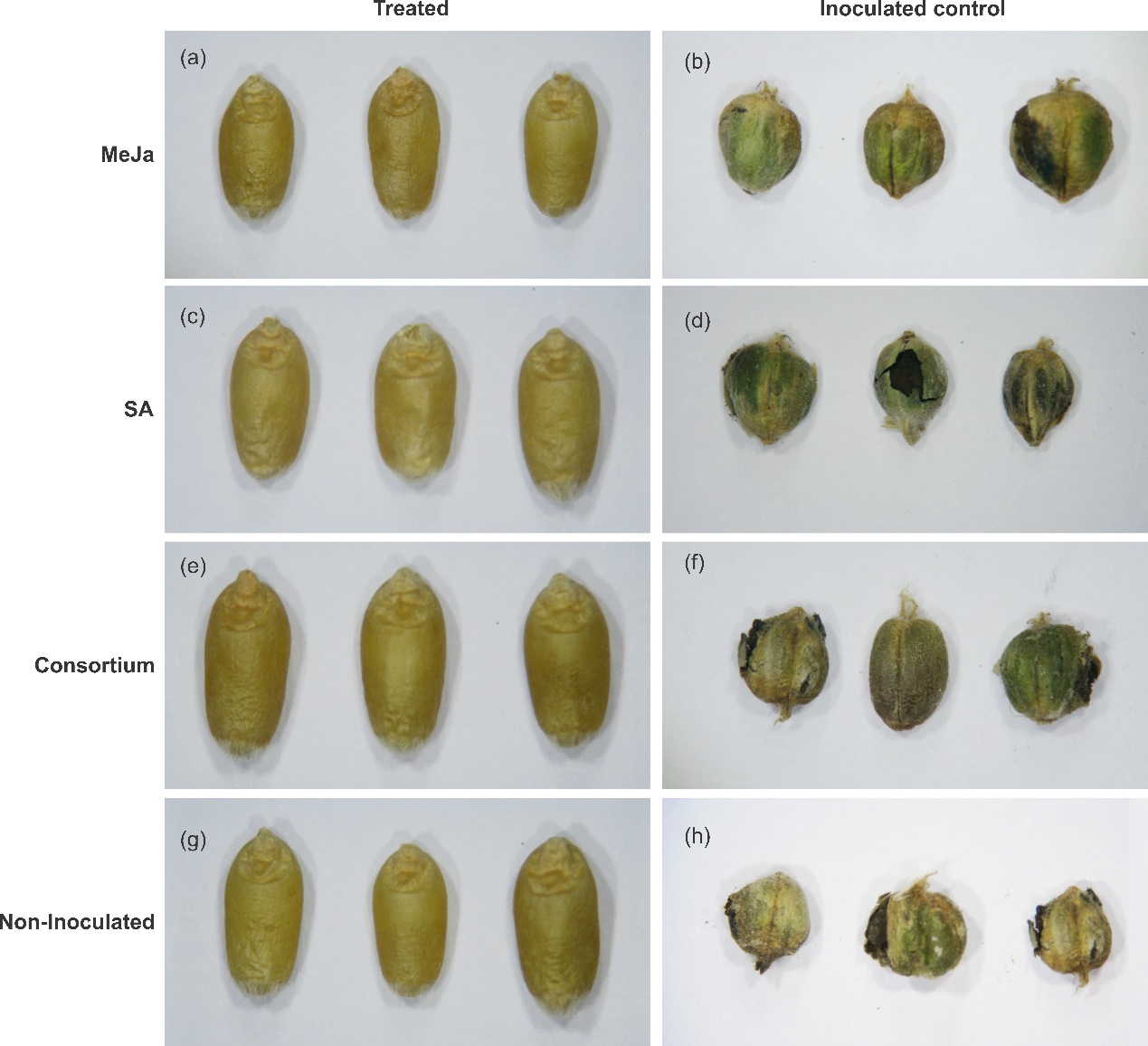


**Figure S3**. Compared seed of treated inoculated, control and non-treated inoculated for disease symptoms. *(a, c, e, g)* No disease symptoms seen in the treated inoculated and control seeds. *(b, d, f, h)* Clear bunt symptoms were observed in the non-treated inoculated seeds.


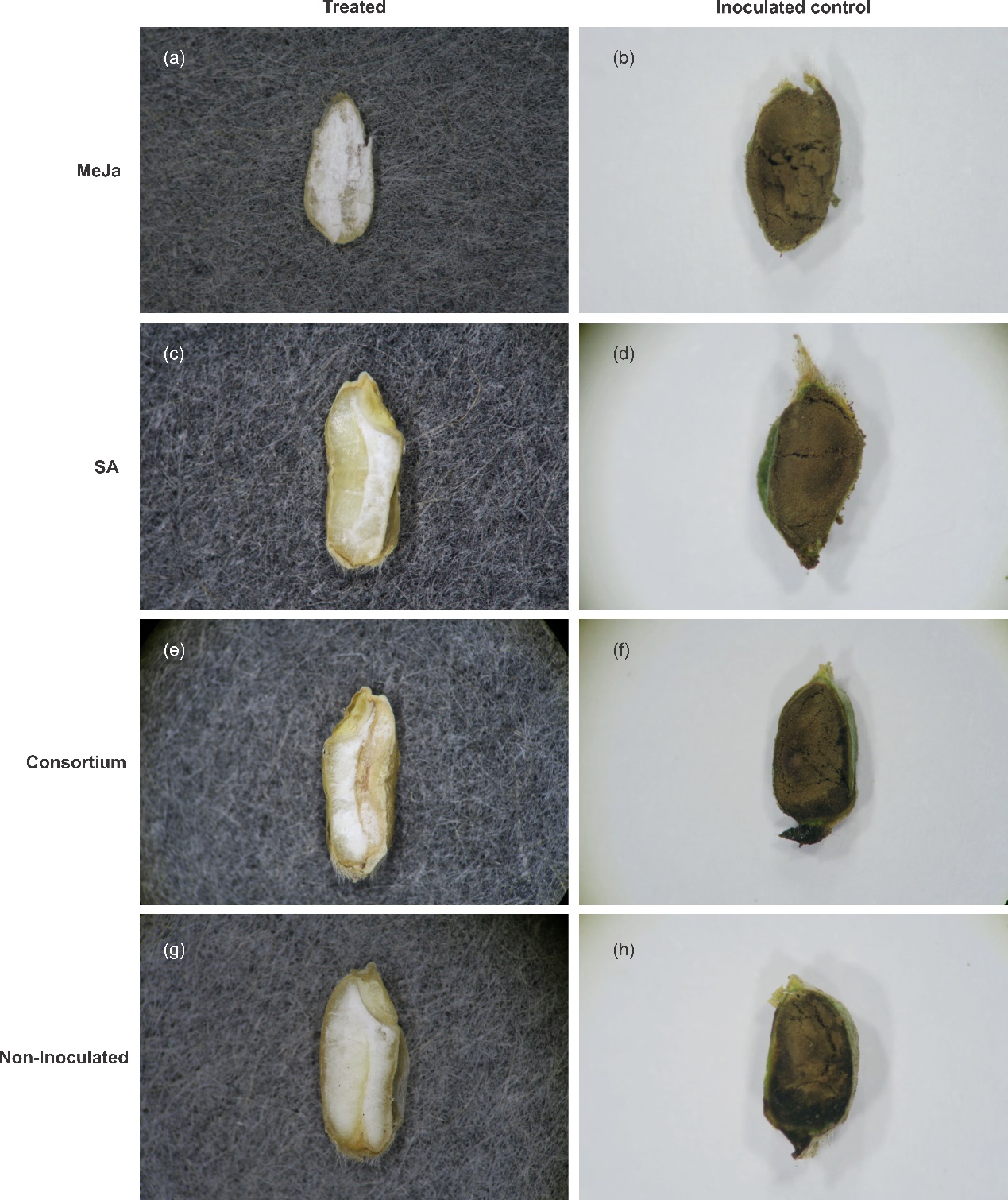


**Figure S4.** Treated inoculated, non-inoculated and inoculated control seeds were cut longitudinally for purpose to look black powder (teliospores) inside the seeds. *(a, c, e, g)* White brown powder were seen in the treated inoculated and non-inoculated seeds. (*(b, d, f, h)* Black powder (teliospores) were seen inside the inoculated control seeds, which contain millions of *T. controversa* teliospores.

**Table S1.** Primers used in this experiment.

| Primer Name | Sequence (5’-3’) | Usage |
| --- | --- | --- |

GPDH 43 F CTCCAACGCTAGTTGC qPCR

GPDH 43 R AACTTTCCGTTAAGCTCTG

COI1-1 F CGGAGGTTCGCTCTATAC qPCR

COI1-1 R CCGACGTTACCCAGAAG

COI1-2 F GCAGGTAACTGATCTGCCACTC qPCR

COI1-2 R GTGCATCCTATCGCAAAGCG

HRin 1 F GTTGCTCTGCTGTGTACAAG qPCR

HRin 1 R GTCCTTTGTCTTTGTCTCAC

PR-10a F CAGCTCAAGTCGCAGGTG qPCR

PR-10a R CTCTCGTACTCCACCCTGAG

Catalase F CCATGAGATCAAGGCCATCT qPCR

Catalase R ATCTTACATGCTCGGCTTGG
